# Prognosis Biomarkers of Severe Sepsis and Septic Shock by ^1^H NMR Urine Metabolomics in the Intensive Care Unit

**DOI:** 10.1371/journal.pone.0140993

**Published:** 2015-11-13

**Authors:** Monica Garcia-Simon, Jose M. Morales, Vicente Modesto-Alapont, Vannina Gonzalez-Marrachelli, Rosa Vento-Rehues, Angela Jorda-Miñana, Jose Blanquer-Olivas, Daniel Monleon

**Affiliations:** 1 Department of Critical Care, Clinical University Hospital of Valencia, Valencia, Spain; 2 Central Unit of Research in Medicine, University of Valencia, Valencia, Spain; 3 Department of Paediatric Critical Care, University and Polytechnic Hospital La Fe, Valencia, Spain; 4 Clinical Hospital Research Foundation-INCLIVA, Valencia, Spain; The Norwegian University of Science and Technology (NTNU), NORWAY

## Abstract

Early diagnosis and patient stratification may improve sepsis outcome by a timely start of the proper specific treatment. We aimed to identify metabolomic biomarkers of sepsis in urine by ^1^H-NMR spectroscopy to assess the severity and to predict outcomes. Urine samples were collected from 64 patients with severe sepsis or septic shock in the ICU for a ^1^H NMR spectra acquisition. A supervised analysis was performed on the processed spectra, and a predictive model for prognosis (30-days mortality/survival) of sepsis was constructed using partial least-squares discriminant analysis (PLS-DA). In addition, we compared the prediction power of metabolomics data respect the Sequential Organ Failure Assessment (SOFA) score. Supervised multivariate analysis afforded a good predictive model to distinguish the patient groups and detect specific metabolic patterns. Negative prognosis patients presented higher values of ethanol, glucose and hippurate, and on the contrary, lower levels of methionine, glutamine, arginine and phenylalanine. These metabolites could be part of a composite biopattern of the human metabolic response to sepsis shock and its mortality in ICU patients. The internal cross-validation showed robustness of the metabolic predictive model obtained and a better predictive ability in comparison with SOFA values. Our results indicate that NMR metabolic profiling might be helpful for determining the metabolomic phenotype of worst-prognosis septic patients in an early stage. A predictive model for the evolution of septic patients using these metabolites was able to classify cases with more sensitivity and specificity than the well-established organ dysfunction score SOFA.

## Introduction

Sepsis is one of the most prevalent diseases and a main cause of death among hospitalized patients in all around the world. In Europe, severe sepsis affects 90.4 cases per 100 000 adult residents per year and an overall hospital mortality of 36% described in the last Sepsis Occurrence in Acutely ill Patients (SOAP) study [1].

Early diagnosis and patient stratification may improve sepsis outcome by a timely start of the proper specific treatment. Sepsis resuscitation and management bundles implementation within the first 24 hours demonstrated better chances of survival [2]. However, the early assessment of severity in sepsis is complicated due to the highly variable and non-specific symptoms and signs. One of the most accepted organic dysfunction scores in sepsis management is the Sequential Organ Failure Assessment score (SOFA) [3]. Although the original design did not include mortality prediction, SOFA has become a useful tool for this purpose. However, the different studies on the subject do not provide sufficient evidence for supporting individual decision-making [4]. Current research on sepsis is oriented on biomarkers for the assessment of the severity of sepsis at an early stage. A recent review study of the subject showed that although up to 178 different molecules have been proposed as potential sepsis biomarkers, none had sufficient specificity or sensitivity to be routinely employed in clinical practice [5]. Combining information collected from several biomarkers, better than from a single molecule, and adding information about the cellular response may be a further approach to help optimize the current anti-infective strategies [6–7]. The application of Nuclear Magnetic Resonance (NMR) metabolomics in critical septic patients would result in the simultaneous identification of a vast array of potential new biomarkers. Moreover, the disease is described by a set of metabolites levels that are involved in septic processes, obtaining a patient’s molecular phenotype “snapshot” of the multiparametric organic and cellular response in sepsis. Differential metabolic signatures at early sepsis stages may be predictive of disease severity. Changes over time of this metabolomic phenotype may be a useful tool for targeting therapy, monitoring therapeutic response, and disease progression [8–11].

The aim of the present study was to identify metabolomic biomarkers of sepsis in urine by ^1^H NMR spectroscopy to assess the severity and to predict outcomes.

## Material and Methods

### Patient enrolment

A prospective observational cohort study was performed in the Intensive Care Unit (ICU) at the Clinical University Hospital of Valencia (Spain). The study was approved by local Ethics Committee, and informed consent forms were signed by all of the subjects prior to participation in this study. Patients were treated according to the rules of the Surviving Sepsis Campaign 2012 [12]. All patients admitted to the ICU who met the following criteria were eligible for the study: diagnosis of severe sepsis or septic shock according to the criteria of Consensus Conference 2001 [13], age between 18 and 85 years-old, without cardiopulmonary resuscitation (CPR), emergency origin, non-surgical, non-pregnant and non-chronic kidney disease.

### Sample collection

Demographic variables related to course of illness and outcomes were collected as part of the daily clinical routine for each patient. SOFA score at admission (SOFA-0h), at 24 hours (SOFA-24h) and at 72 hours (SOFA-72h) after admission to the ICU were evaluated for each patient according the protocol established in the ICU and published criteria for SOFA [3]. All patients were followed up for 30 days after enrolment in the study in order to obtain data about 30-days mortality. Urine-0h samples were collected from the first urine after catheterization for each patient on the day of admission. Twenty-four hours after admission in the ICU, the urinary catheter was blocked to collect the second urine sample (Urine-24h). Immediately after collection, samples were frozen at—80°C until the NMR measurements.

### 1H NMR Spectroscopy and metabolite concentration profiling

Forty microliters of a phosphate buffer solution (0.06M Na_2_HPO_4_/ 0.04M NaH_2_PO_4_, pH 7) and 40μl of sodium-3´-trimethylsilylpropionate-2,2,3,3-d_4_ (TSP, 0.5 mM) in deuterium oxide were mixed with 420μl of urine and placed in a 5mm high-resolution NMR tube. NMR spectra were acquired using a standard one-dimensional pulse sequence with water suppression in a Bruker Avance DRX 600MHz spectrometer (Bruker Biospin GmbH, Rheinstetten, Germany). A total of 64 FIDs (free induction decay) were collected into 64k data points with a spectral width of 14 ppm at 310K.

Water pre-saturation for 1 s along the recycling delay was used for solvent signal suppression. The spectral width for all spectra was 14 ppm for 1H. Before Fourier transformation, the free induction decay was multiplied by a 0.3 Hz exponential line broadening. All spectra were phased, baseline corrected carefully and chemical shifts were adjusted with reference to TSP signal using MestRenova 6.2 software (Mestrelab Research S.L., Santiago de Compostela, Spain). The spectra were binned into 0.005 ppm buckets between 0.5–10 ppm and mean centered for multivariate analysis and normalized to total aliphatic spectral area (0.5–4.4 ppm) to eliminate differences in metabolite total concentration. Data were imported into MATLAB R2012a (The MathWorks Inc., Natick, MA 2012) for additional processing and further analysis. Signals belonging to selected metabolites were integrated and quantified using semi-automated in-house MATLAB peak-fitting routines. Resonances were assigned according to the previous literature [14] and the Human Metabolome Database (http://www.hmdb.ca).

### Statistical Analysis

Chemometrics statistical analyses were performed using in-house MATLAB scripts and the PLS Toolbox 6.7 (Eigenvector Research, Inc., Wenatchee, WA, USA). Metabolite levels were expressed as mean ± SD (standard deviation). One-way-analysis of variance (ANOVA) was used in order to determinate the statistical significance between the means in both survivor and non-survivor groups. Principal component analysis (PCA) and projection to latent structures for discriminant analysis (PLS-DA) were applied to NMR spectral datasets. Results were cross-validated using the leave-one-out to evaluate the accuracy of each classification model [15]; in each run one sample of the data is left out of the training and used to test the model. The whole cross validation process was run 10 times. The results of cross validation were evaluated by the Q2 (R2CV) and RMSCV parameters. Q2 is the averaged correlation coefficient between the dependent variable and the PLS-DA predictions and provides a measure of prediction accuracy during the cross-validation process (higher values mean better prediction). Root Mean Square Error of Cross-Validation (RMSCV) was calculated as an adequate measurement of over fitting. Permutation test was done for testing for over-fit regression models (Random t-test) as well as for providing a probability that the given model is significantly different from one built under the same conditions but on random data. To compare performance in the mortality prediction of metabolomic and SOFA scores a multi-step statistical analysis was performed based on the study of Cabré et al [16]. Goodness of the diagnostic method was evaluated using a ROC curve based on the logistic regression data and calculating the diagnostic performance indexes. Additional detail about NMR data processing and the construction and validation of multivariable metabolomic models is provided in the Supporting Information section.

## Results

A total of 64 patients were enrolled during the study period. Clinical and demographic characteristics of the patients are shown in Table 1. Half of the episodes were diagnosed as severe sepsis and half septic shock. The average age of patients was 60 (interquartile range, 47–73) of which 65% were male, with an average ICU stay of 7 days (interquartile range, 4–11) and a 30-days mortality of 20% (interquartile range, 9.2–29.6). At the first day, the average APACHE II (Acute Physisology and Chronic Health Evaluation II) and SOFA scores were 19.6 (± 6.03) and 7.8 (± 3.6), respectively. The predominant sites of initial infection were lungs (66.7% of cases), abdomen (13.3%), urinary tract (13.3%) and central nervous system (6.7%). Blood culture was positive in 22 patients (36.7%). Typical examples of ^1^H NMR urinary spectra from survivor and non-survivor patients are shown in S1 Fig.

**Table 1:** Demographic and clinical characteristics of the subjects enrolled in the study.

Items	All	Survivor	Non- survivor	*p value
	(n = 60)	(n = 48)	(n = 12)	
Male sex, n (%); [IC]	39 (65%); [53.6–77.8]	32 (66.7%)	7 (58.3%)	ns
Age, years, median (IQR); [IC]	60 (47–73); [55.3–62.8]	60 (24–80)	65 (37–79)	ns
Severe sepsis, n (%); [IC]	30 (50%); [38–63.5]	27 (56.2%)	3 (25%)	ns
Septic shock, n (%); [IC]	30 (50%); [36.5–62]	21 (43.8%)	9 (75%)	ns
Days in the ICU, median (IQR); [IC]	7 (4–11); [5–9]	6.5 (4.7–11)	7 (3–22.5)	ns
30-day mortality, n (%); [IC]	12 (20%); [9.2–29.6]			
APACHE II, mean ± SD; [IC]	19.6 ± 6.0; [18.1–21.1]	19 ± 6	21 ± 5	ns
SOFA-0h, median (IQR)	8 (6.8–8.6)	7 (5–10)	11 (7–13.5)	< 0.05
SOFA-24h, median (IQR)	5 (3–8)	5 (3–7)	8 (4.75–9.75)	< 0.05
SOFA-72h, median (IQR)	3 (2–6)	3.5 (2–5)	6.5 (2–10.25)	< 0.05
Origin of sepsis, n (%); [IC]:				
Abdominal	7 (11.7%); [2.4–18]	6 (12.5%)	1 (8.3%)	ns
Pulmonar	38 (63.3%); [52–76.4]	31 (64.6%)	7 (58.3%)	ns
Urinary	8 (13.3%); [4.5–22.3]	7 (14.6%)	1 (8.3%)	ns
CNS	4 (6.7%); [1.7–14.6]	4 (8.3%)	0	ns
Bacteremia, n (%); [IC]	22 (36.7%); [23.6–48]	14 (29.2%)	8 (66.7%)	<0.05
Analytic results:				
Hemoglobin (g/dl), mean ± SD; [IC]	11.1 ± 1.7; [10.6–11.5]	11.1 ± 1.8	9.8 ± 1.8	<0.05
Leukocytes (x10^9^/l), mean ± SD; [IC]	14.8 ± 10.7; [10.3–17.5]	15.92 ± 11.8	10.2 ± 9.6	ns
Platelets (x10^9^/l, mean ± SD; [IC]	193 ± 94; [169–230]	207 ± 105	135 ± 94	<0.05
Glucose (mg/dl), median (IQR); [IC]	140 (107–175); [127–151]	139 (107–168)	134 (109–212)	ns
Bilirubin (mg/dl), median (IQR); [IC]	0.9 (0.5–1.4); [0.6–1.1]	1.2 ± 1.3	1.13 ± 5.1	ns
PCR (mg/l), mean ± SD; [IC]	172 ± 46; [134–182]	174 ± 87	162 ± 76	ns
PCT (ng/ml), median (IQR); [IC]	3.9 (0.6–20); [1.4–9.8]	2.5 (0.5–16.9)	5.7 (0.8–44.8)	ns
Lactate (mmol/l), median (IQR); [IC]	2.6 (1.7–3.6); [2.3–3.1]	2.6 (2.0–3.5)	2.75 (1.6–7.8)	ns
Creatinine (mg/dl), median (IQR); [IC]	1.6 (0.9–2.5); [1.2–2]	1.5 (0.9–2.2)	1.3 (0.8–2.6)	ns
Urea (mg/dl), mean ± SD; [IC]	74 ± 46, [61.9–91.3]	70 ± 4	89 ± 55	ns

*ICU* intensive care unit, *APACHE II* Acute Physiology and Chronic Health Evaluation II, *SOFA* Sequential Organ Failure Assessment, *PCT* procalcitonin, *PCR* reactive C protein, *CNS* central nervous system, *SD* standard deviation, *IQR* interquartile range, *IC* confidence interval 95%;

*p values, survivor vs non-survivor; ns, no significant value (p ≥ 0.05).

**Fig 1:**
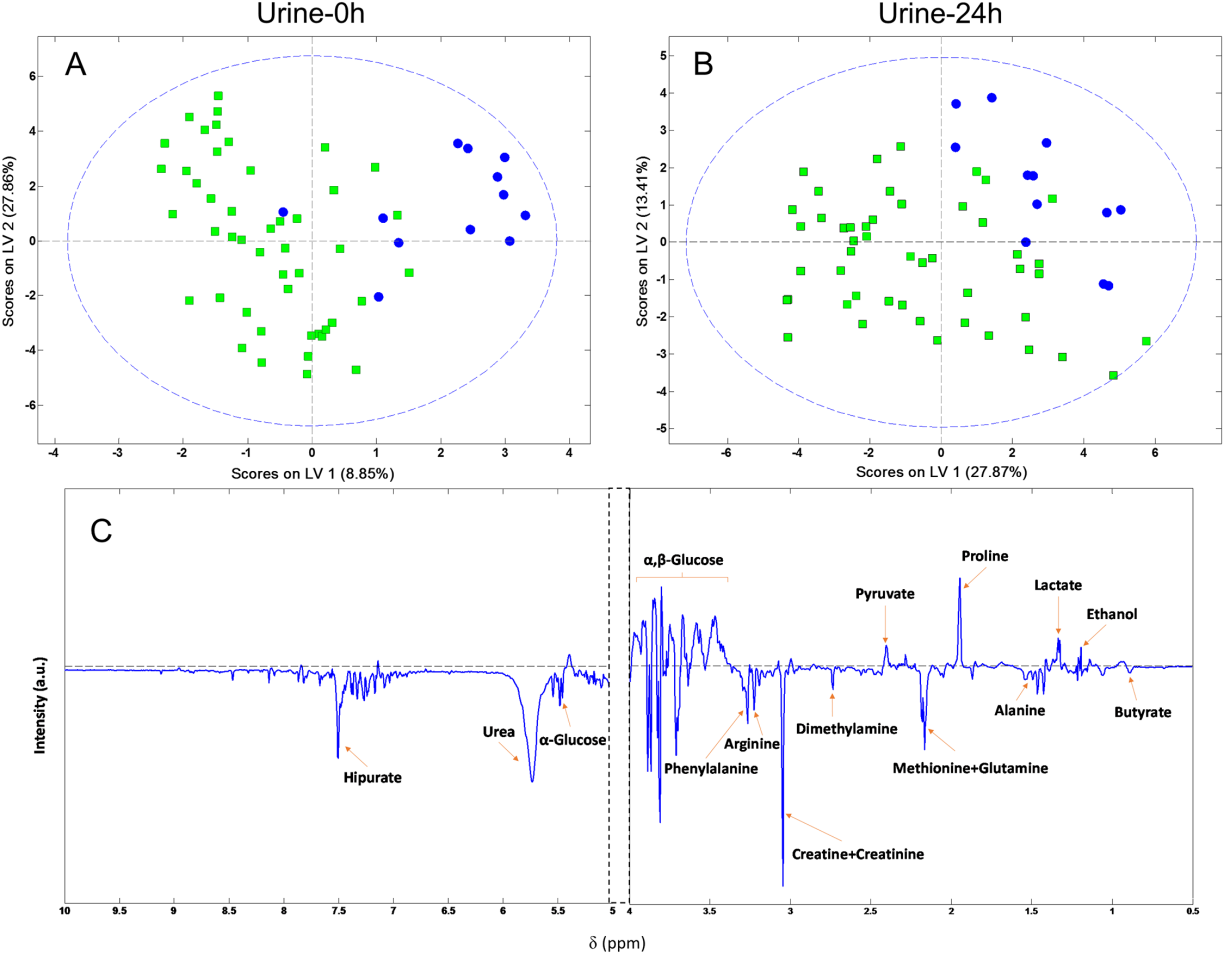
PLS-DA score plot for discrimination between survivor (open squares) and non-survivor patients (black circles). (A) PLS-DA score plot constructed with Urine-0h samples obtained at the admission to the ICU. (B) PLS-DA score plot constructed with Urine-24h samples obtained at 24h after admission to the ICU. (C) Loading plot of the PLS-DA score plot constructed with Urine-0h samples.

Based on the PCA analysis, four samples were identified as outliers and excluded for further statistical analysis (one sample in the non-survivor group and three samples in the survivor group). PLS-DA was performed to reveal specific metabolic changes in defined groups and improve the separation between patients. Two independent discriminating models (based on Urine-0h and -24h) were constructed. The score scatter plot of the PLS-DA model built from Urine-0h and Urine-24h samples are presented in Fig 1. An excellent separation with minimal overlapping was observed between positive and negative prognosis in the Urine-0h model. Separation in two groups resulted even more evident in the discriminating model based on the Urine-24h samples. Furthermore, this separation is described with high values of area under the ROC curve as 0.83 (CI 0.71–0.95) and 0.88 (CI 0.78–0.97) for Urine-0h and -24h models, respectively. In addition, high values of the model’s goodness-of-fit metrics (R2Y = 0.84, Q2 = 0.44 and R2Y = 0.81, Q2 = 0.43 for Urine-0h and -24h, respectively) indicated robustness and reproducibility (S2 Fig).

**Fig 2:**
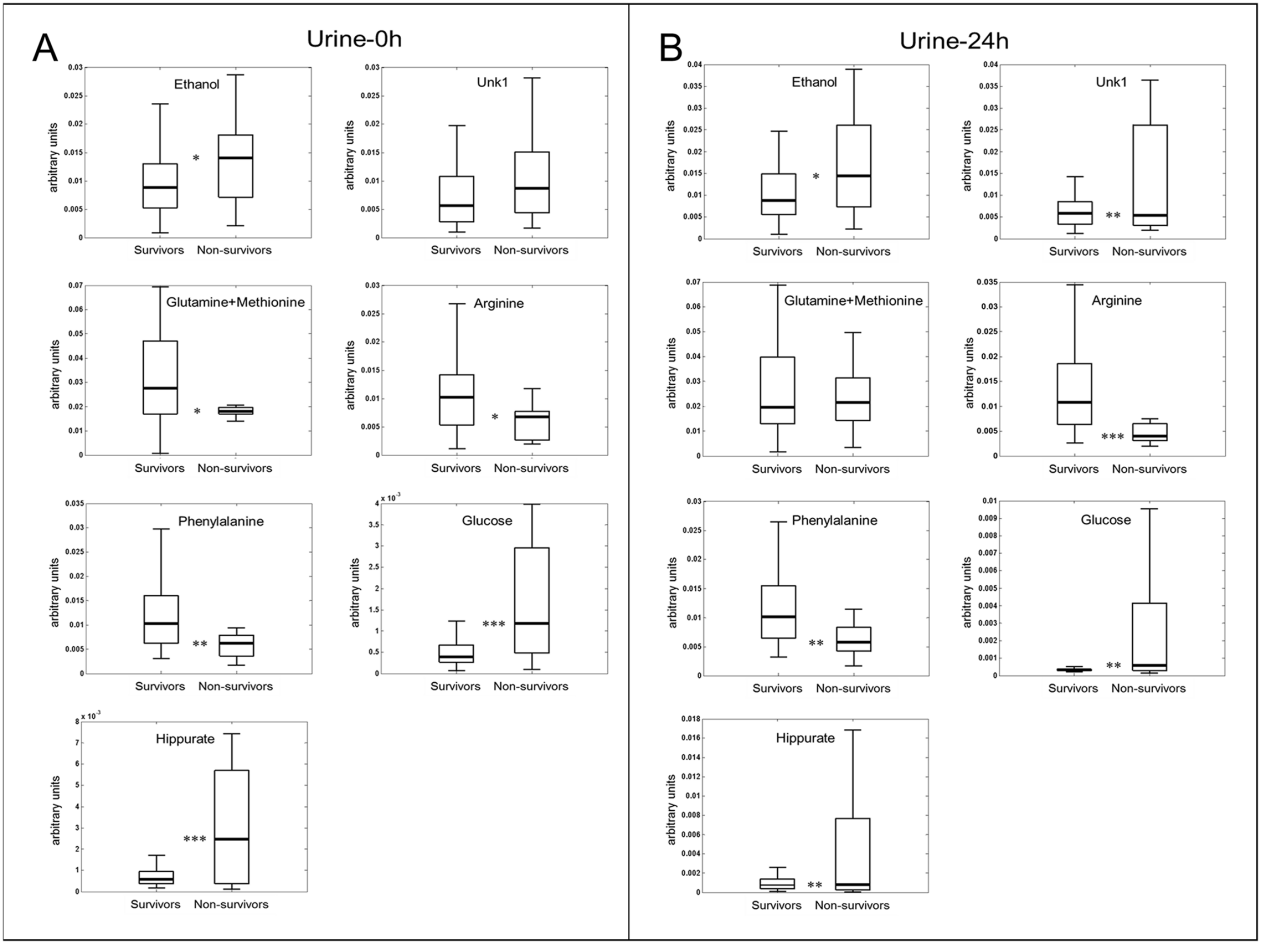
Box plots showing representative metabolite changes between survivor and non-survivor patients. (A) Box plots from Urine-0h (B) Box plots from Urine-24h samples. Boxes denote interquartile ranges, lines denote medians, and whiskers denote 10th and 90th percentiles. Levels are expressed as area of the metabolite of interest divided with respect total aliphatic spectral area.*p < 0.05; **p < 0.01;***p < 0.001.

Specific metabolites that had major contribution to this metabolic differential pattern were identified from the PLS-DA analysis and subsequently, the area of these regions was integrated. Mean and standard deviation of metabolites, at the day of admission and at the second day in the ICU, with significant changes in concentration (p <0.05) were considered (S1 Table). The assignation of the peak included in the region between 1.40–1.45 ppm remained inconclusive to any known metabolite. Negative prognosis patients presented higher values of ethanol, glucose, hippurate and an unknown compound, but lower levels of methionine, glutamine, arginine and phenylalanine. Box-plots of those metabolites that set the differential profile pattern at the day of admission and 24-hours later, respectively, are shown in Fig 2.

**Fig 3:**
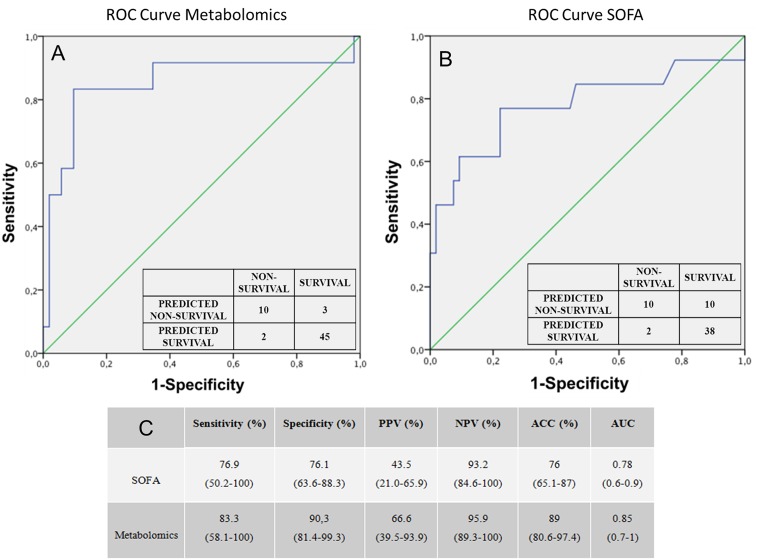
ROC curves constructed with the logistic regression model and frequency table for discrimination between survivor and non-survivor patients in predicting 30-days mortality. (A) ROC curve based on the metabolomics scores evolution values (AUC 0.85; p<0.05, 95% CI 0.68–1). (B) ROC curve based on the SOFA evolution values (AUC 0.78; p<0.05, 95% CI 0.6–0.9). (C) Comparison of sensitivity, specificity, positive predictive value (PPV), negative predictive value (NPV), accuracy (ACC) and area under (AUC) the ROC curve of SOFA and metabolomic predictive models. The numbers in parenthesis represent the confidence interval in 95%.

In order to make comparable the death prediction value of metabolomic model and SOFA score a multi-step statistical analysis was performed. The logistic regression ROC curve obtained with the metabolomic values (Fig 3A) has an AUC of 0.85 (CI 0.69–1)). Whereas the ROC curve obtained with the logistic regression SOFA values (Fig 3B) has an AUC of 0.78 (CI 0.70–0.95). Sensitivity, specificity, positive and negative predictive values and its corresponding confidence interval of 95% for both ROC curves are summarized in Fig 3C. The urine-based metabolomic score obtained from the logistic regression performed better discrimination between positive and negative prognosis than with SOFA scale. As the number of samples in the survivor and non-survivor groups was not well balanced, we performed a new model with a subset of samples from survivals matched with respect gender, age, SOFA and Bacteremia of the non-survival group (see S3 Fig). This model did not exhibit better discriminating results than the obtained with non-matched samples.

An individualized analysis of cases where a significant disparity existed between the prediction of SOFA scale and the metabolomic score confirmed the good results of the metabolomic predictive model. In two types of patients, metabolomics approach seems to be more efficient than the SOFA scores in the early prognosis of death. Patient type 1, with SOFA values of equal or less than 8 but with a high probability of worse prognosis based on its metabolomic scores, had a final negative evolution in agreement with metabolomic prediction. Patient type 2, with poor initial SOFA prognosis values that decreased quickly in the first 24h but metabolomics scores predicting a high probability of death during the evolution, had a fatal outcome. In our study no cases were found of deceased patients with an initial high SOFA value and an opposite metabolomic evolution prediction. All available data for assessment of the prognosis of these particular cases are summarized in the S2 Table.

## Discussion

The daunting challenge that bedside critical care practitioners face daily is how to identify the septic worse prognosis patients rapidly and with precision. Biomarkers use in critically ill patients is recognized as essential adjuncts in this process. The complex pathophysiology of sepsis suggests that a single biomarker approach cannot adequately describe and stratify the septic syndrome. Probably this is one of the reasons why neither clinical nor biological biomarkers have shown to be highly accurate to predict adverse outcomes. To our knowledge, this is the first study in adults that describe a metabolic model prediction of mortality in septic patients using ^1^H NMR spectroscopy in urine samples.

The metabolic profile differences at first 24 hours between survivor/non-survivor groups allowed the selection of 8 metabolites (ethanol, glutamine, methionine, arginine, phenylalanine, glucose, hippurate and an unknown metabolite located at 1.40–1.45 ppm) that all together constitute a biosignature with potential prognostic value. Identified metabolites are involved in several biochemical alterations typically described in septic patients including protein levels, fatty acids synthesis, energy metabolism deregulation and even indicators of microbiota disruption. Arginine intervenes in the synthesis of acute phase proteins in severe infection, as well as in the nitric oxide synthesis at the vascular level. A decrease of its levels in patients with severe sepsis has been previously described [17–18]. Glutamine plays a role in the fatty acid and protein synthesis, and it is a primary energy source for many cells such as the intestinal epithelial, immune system and cardiac cells. Their values are diminished in severe sepsis, which in turn is associated with an increased mortality [19]. Alterations in the energy metabolism are reflected clearly in altered glucose levels. Stress hyperglycaemia is usually associated with an injury, inflammation process or severe infections. It occurs because of glycogenolysis in muscle and liver, hepatic gluconeogenesis and insulin resistance by peripheral tissues. In addition, hyperglycaemia in critically ill patients is a prognostic predictor of mortality during the disease process [20]. Hippurate urinary levels are associated with the intestinal microbial profile as an indirect indicator of microbiota alterations. The gut microbial activity is contributing to the difference in the metabolic processing of polyphenolic compounds within the colon, like the hippurate and, consequently, could explain the differences of the microbial-derived urinary metabolites. The antibiotic administration induces the suppression of the gut microbiota and it results in a reduction in the excretion of hippurate levels [21]. Our results show that in patients with poor prognosis this reduction is not as pronounced as in patients with good prognosis. The antibiotic therapy was confirmed to be the best suited for each patient by expert microbiologists. Therefore, we may assume that the association between poor prognosis and higher excretion of hippurate did not reflect a deficient empirical antibiotic strategy. Phenylalanine is typically associated with increased muscle catabolism. As far as we know, there is no published evidence of any relation between phenylalanine levels in urine and sepsis process. However, elevated levels in plasma and cerebrospinal fluid have been found in patients with septic encephalopathy [22]. Finally, although changes in ethanol could have been related to different dietary habits, these differences persisted after 24 hours of admission to the hospital. At this moment, patients are fasting or have a standardized diet, thus suggesting an endogenous alcohol-producing microbiota origin. In fact, it has been reported that members of the genus Escherichia are associated with high blood ethanol concentrations, which in turn is associated with an increase of gut permeability and inflammation [23]. In addition, elevated ethanol production may suggest a switch of the glycolysis to a fermentative metabolism which is maintained through the alcohol dehydrogenase activity (LDH). It has been reported the essentiality of LDH in the virulence of fermentative microorganisms that causes bacteremia like *Streptococcus pneumoniae* [24], *Staphylococcus aureus* [25] or *Enterococcus faecalis* [26].

Classical biomarkers have not been shown to be better than generic scores in predicting outcome in septic patients [27]. Based on the results obtained in the first 24 hours, we have established the characteristic metabolic phenotype of a septic patient with a good performance against the worst prognosis as a combination of eight urine metabolite levels. Using these metabolites, a predictive model of outcome has been constructed. From this model, we may estimate a metabolomic score that could be interpreted as a probability of worse prognosis. We considered that a combination of several molecules might be more effective than a single one because a wide range of pathophysiological information is included.

We found that our metabolomic predictive model perform better than the commonly used SOFA score in predicting short-term outcome in severe sepsis or septic shock. These results are in accordance with a recently publication of paediatric septic patients serum study [28]. In this study, a metabolomic non-survivor predictive model was compared to the Paediatric Risk of Mortality III-Acute Physiology Score (PRISM III-APS). They concluded that their metabolomic model was better in early identification of bad prognosis. The same research group conducted a study with serum from adult patients diagnosed with septic shock compared with systemic inflammatory response syndrome (SIRS) that allowed them to predict mortality better than the APACHE II and SOFA scales [29]. In addition, we observed that our metabolomic predictive model needs shorter evolution period (24h instead of 72h) than SOFA score to reach predictive values. A similar conclusion was reached in an experimental sepsis model in rats where authors found that changes in serum metabolic profiles occurred earlier than those of organ dysfunction [30].

Septic patient studies have been usually performed on blood samples. The urinary metabolites studies that use urine samples to measure metabolites related with infection are scarce [31]. Given the high prevalence of anaemia in critically ill patients and the negative influence of repeated blood sampling in clinical routine, we wanted to test urine samples to avoid this secondary effect. Although metabolomic profile in urine may be more influenced, compared to serum, by other factors not related to sepsis (diet, time of sampling, some drugs…), the use of powerful multivariate Chemometrics techniques could overcome these limitations. Another limitation is that a high percentage of severe septic patients show renal failure and oligoanuria. This could be an obstacle for obtaining enough sample volume for metabolomic analysis. However, NMR measurements only require 0.5 ml of urine. On the other hand, the metabolite differences observed in urine samples could be attributed precisely to the renal failure rather than to the sepsis. However, no significant differences in creatinine and urea levels were observed between the non-survivor and survivor groups. We developed a model with potential clinical application using a type of sample easy to obtain in all clinical settings. Undoubtedly, the clinical validation of the developed metabolic biosignature requires further studies including large series of patients.

The future trend of Medicine is trying to get personalized treatments. An individualized initial metabolic profile and its evolution could play a major role for targeting appropriate drugs, doses and moment to initiate the therapy. Metabolomics could contribute significantly in the desired process of implementation of personalized medicine due to metabolomic disorders detected in urine precede organic failures that will be subsequently collected by SOFA scale.

## Conclusion

In conclusion, urine NMR metabolomics analysis can be a useful tool to describe and interpret the pathophysiological processes that occur in sepsis. Metabonomics could contribute significantly in the desired process of implementation of personalized medicine due to metabonomic disorders detected in urine precede organic failures that will be subsequently collected by SOFA scale. For these reasons, guided by judicious clinical assessment, NMR metabonomics could be a complementary tool to classical methods used until now to stratify better the prognosis in septic shock and severe sepsis. Promising results and its numerous advantages (non-invasive, cheap, fast and low-cost measure), suggest that NMR metabonomics could be included in the clinical routine in the near future.

## Supporting Information

S1 Fig Average 1H NMR spectrum of urine-0h samples obtained at the admission to the ICU from septic patients. Comparison between survivor (green) and non-survivor (blue) patients’ metabolomic profile and assignation of different metabolites.
(TIF)

S2 Fig ROC curves for discrimination between survivor and non-survivor patients constructed with A) Urine-0h and B) Urine-24h samples based on our PLS-DA model (blue line, training data; green line, leave-one-out cross-validation). (TIF)

S3 Fig PLS-DA score plot for discrimination between survivor (green squares) and non-survivor patients (blue circles) matched by age, gender, SOFA and bacteremia. A) PLS-DA score plot constructed with Urine-0h samples obtained at the admission to the ICU and B) PLS-DA score plot constructed with Urine-24h samples obtained at 24h after admission to the ICU. ROC curves for discrimination between survivor and non-survivor patients constructed with C) Urine-0h and D) Urine-24h samples based on the matched PLS-DA model (blue line, training data; green line, leave-one-out cross-validation).
(TIF)

S1 Table (DOCX)

S2 Table (DOCX)

S3 Table (DOCX)

S1 Text (DOCX)

## References

[1] Vincent JL, Sakr Y, Sprung CL, Ranieri VM, Reinhart K, Gerlach H, et al Sepsis in European intensive care units: results of the SOAP study. Crit Care Med 2006; 34: 344–53. 16424713 10.1097/01.ccm.0000194725.48928.3a

[2] Rivers EP, Ahrens T. Improving outcomes for severe sepsis and septic shock: tools for early identification of at-risk patients and treatment protocol implementation. Crit Care Clin 2008; 24: S1–47. 10.1016/j.ccc.2008.04.002 18634996

[3] Vincent JL, Moreno R, Takala J, Willatts S, De Mendoça A, Bruining H, et al The SOFA (Sepsis-related Organ Failure Assessment) score to describe organ dysfunction/failure. On behalf of the Working Group on Sepsis-Related Problems of the European Society of Intensive Care Medicine. Intensive Care Med 1996; 22: 707–10. 8844239 10.1007/BF01709751

[4] Minne L, Abu-Hanna A, de Jonge E. Evaluation of SOFA-based models for predicting mortality in the ICU: A systematic review. Crit Care 2008; 12: R161 10.1186/cc7160 19091120 PMC2646326

[5] Pierrakos C, Vincent JL. Sepsis biomarkers: a review. Crit Care 2010; 14: R15 10.1186/cc8872 20144219 PMC2875530

[6] Vincent JL, Teixeira L. Sepsis biomarkers value and limitations. AJRCCM 2014; 190:1081–2. 10.1164/rccm.201410-1895ED 25398103

[7] Bodi V, Sanchis J, Morales JM, Marrachelli VG, Nunez J, Forteza MJ, et al Metabolomic profile of human myocardial ischemia by nuclear magnetic resonance spectroscopy of peripheral blood serum. A translational study based on transient coronary occlusion models. J Am Coll Cardiol 2012; 59: 1629–41. 10.1016/j.jacc.2011.09.083 22538333

[8] Serkova NJ, Standiford TJ, Stringer KA. The emerging field of quantitative blood metabolomics for biomarker discovery in critical illnesses. Am J Respir Crit Care Med 2011; 184: 647–55. 10.1164/rccm.201103-0474CI 21680948 PMC3208597

[9] Skibsted S, Bhasin MK, Aird WC, Shapiro NI. Bench-to-bedside review: Future novel diagnostics for sepsis—a systems biology approach. Crit Care 2013; 17: 231 10.1186/cc12693 24093155 PMC4057467

[10] Noto A, Mussap M, Fanos V. Is 1H NMR metabolomics becoming the promising early biomarker for neonatal sepsis and for monitoring the antibiotic toxicity?. J Chemother 2014; 26: 130–2. 10.1179/1973947813Y.0000000149 24112754

[11] Izquierdo-García JL, Naz S, Nin N, Rojas Y, Erazo M, Martínez-Caro L, et al A Metabolomic approach to the pathogenesis of ventilator-induced lung injury. Anesthesiology 2014; 120: 694–702. 10.1097/ALN.0000000000000074 24253045

[12] Dellinger RP, Levy MM, Rhodes A, Annane D, Gerlach H, Opal SM, et al Surviving Sepsis Campaign: international guidelines for management of severe sepsis and septic shock, 2012. Intensive Care Med 2012; 39: 165–228. 10.1007/s00134-012-2769-8 PMC7095153 23361625

[13] Levy MM, Fink MP, Marshall JC, Abraham E, Angus D, Cook D, et al 2001 SCCM/ESICM/ACCP/ATS/SIS International Sepsis Definitions Conference. Crit Care Med 2003; 31: 1250–6. 12682500 10.1097/01.CCM.0000050454.01978.3B

[14] Holmes E, Foxall PJ, Spraul M, Farrant RD, Nicholson JK, Lindon JC. 750 MHz 1H NMR spectroscopy characterisation of the complex metabolic pattern of urine from patients with inborn errors of metabolism: 2-hydroxyglutaric aciduria and maple syrup urine disease. Anal Chem 1995; 67: 793–811. 9260660 10.1016/s0731-7085(97)00066-6

[15] Eriksson L, Johansson E, Kettaneh-Wold N. Multi- and Megavariate Data Analysis Part I: Basic Principles and Applications. Umea, Sweden, MKS Umetrics AB, 2006.

[16] Cabré L, Mancebo J, Solsona JF, Saura P, Gich I, Blanch L, et al Working group of the SEMICYUC. Multicenter study of the multiple organ dysfunction syndrome in intensive care units: the usefulness of Sequential Organ Failure Assessment scores in decision making. Intensive Care Med 2005; 31: 927–33. 15856171 10.1007/s00134-005-2640-2

[17] Luiking YC, Poeze M, Ramsay G, Deutz NE. The role of arginine in infection and sepsis. J Parenter Enteral Nutr 2005; 29: S70–4. 10.1177/01486071050290S1S70 15709548

[18] Luiking YC, Ten Have GA, Wolfe RR, Deutz NE. Arginine de novo and nitric oxide production in disease states. Am J Physiol Endocrinol Metab 2012; 303: E1177–89. 10.1152/ajpendo.00284.2012 23011059 PMC3517635

[19] Wischmeyer PE. Glutamine: mode of action in critical illness. Crit Care Med 2007; 35: S541–4. 17713406 10.1097/01.CCM.0000278064.32780.D3

[20] Griesdale DE, de Souza RJ, van Dam RM, Heyland DK, Cook DJ, Malhotra A, et al Intensive insulin therapy and mortality among critically ill patients: a meta-analysis including NICE-SUGAR study data. CMAJ 2009; 180: 821–7. 10.1503/cmaj.090206 19318387 PMC2665940

[21] Lees HJ, Swann JR, Wilson ID, Nicholson JK, Holmes E. Hippurate: the natural history of a mammalian-microbial cometabolite. J Proteome Res 2013; 12: 1527–46. 10.1021/pr300900b 23342949

[22] Green R, Scott LK, Minagar A, Conrad S. Sepsis associated encephalopathy (SAE): a review. Front Biosci 2004; 9: 1637–41. 14977574 10.2741/1250

[23] Zhu L, Baker SS, Gill C, Liu W, Alkhouri R, Baker RD, et al Characterization of gut microbiomes in nonalcoholic steatohepatitis (NASH) patients: a connection between endogenous alcohol and NASH. Hepathology 2013; 57:601–9. 10.1002/hep.26093 23055155

[24] Gaspar P, Al-Bayati FA, Andrew PW, Neves AR, Yesilkaya H. Lactate dehydrogenase is the key enzyme for pneumococcal pyruvate metabolism and pneumococcal survival in blood. Infect Immun. 2014; 82:5099–109. 10.1128/IAI.02005-14 25245810 PMC4249287

[25] Richardson AR, Libby SJ, Fang FC. A nitric oxide-inducible lactate dehydrogenase enables Staphylococcus aureus to resist innate immunity. Science 2008; 319: 1672–6. 10.1126/science.1155207 18356528

[26] Rana NF, Sauvageot N, Laplace JM, Bao Y, Nes I, Rince A, et al Redox balance via lactate dehydrogenase is important for multiple stress resistance and virulence in Enterococcus faecalis. Infect. Immun 2013; 81:2662–8. 10.1128/IAI.01299-12 23649090 PMC3719593

[27] Antonelli M, Azoulay E, Bonten M, Chastre J, Citerio G, Conti G, et al Year in review in Intensive Care Medicine, 2008: III. Paediatrics, ethics, outcome research and critical care organization, sedation, pharmacology and miscellanea. Intensive Care Med 2009; 35: 405–16. 10.1007/s00134-009-1433-4 19205660 PMC7095358

[28] Mickiewicz B, Vogel HJ, Wong HR, Winston BW. Metabolomics as a novel approach for early diagnosis of pediatric septic shock and its mortality. Am J Respir Crit Care Med 2013; 187: 967–76. 10.1164/rccm.201209-1726OC 23471468 PMC3707368

[29] Mickiewicz B, Duggan GE, Winston BW, Doig C, Kubes P, Vogel HJ. Alberta Sepsis Network. Metabolic profiling of serum samples by ^1^H nuclear magnetic resonance spectroscopy as a potential diagnostic approach for septic shock. Crit Care Med 2014; 42: 1140–9. 10.1097/CCM.0000000000000142 24368342

[30] Lin ZY, Xu PB, Yan SK, Meng HB, Yang GJ, Dai WX, et al A metabolomicapproach to early prognostic evaluation of experimental sepsis by 1H NMR and pattern recognition. NMR Biomed 2009; 22: 601–8. 19322815 10.1002/nbm.1373

[31] Slupsky CM, Rankin KN, Fu H Chang D, Rowe BH, Charles PG, McGeer A, et al Pneumococcal Pneumonia: Potential for Diagnosis through a Urinary Metabolic Profile. J. Proteome Res 2009, 8: 5550–8. 10.1021/pr9006427 19817432

