# Supplementary figures and images for: Prognosis Biomarkers of Severe Sepsis and Septic Shock by 1H NMR Urine Metabolomics in the Intensive Care Unit
Source: PLoS One. 2015 Nov 13;10(11):e0140993. doi: 10.1371/journal.pone.0140993 (PMC4643898; doi:10.1371/journal.pone.0140993)

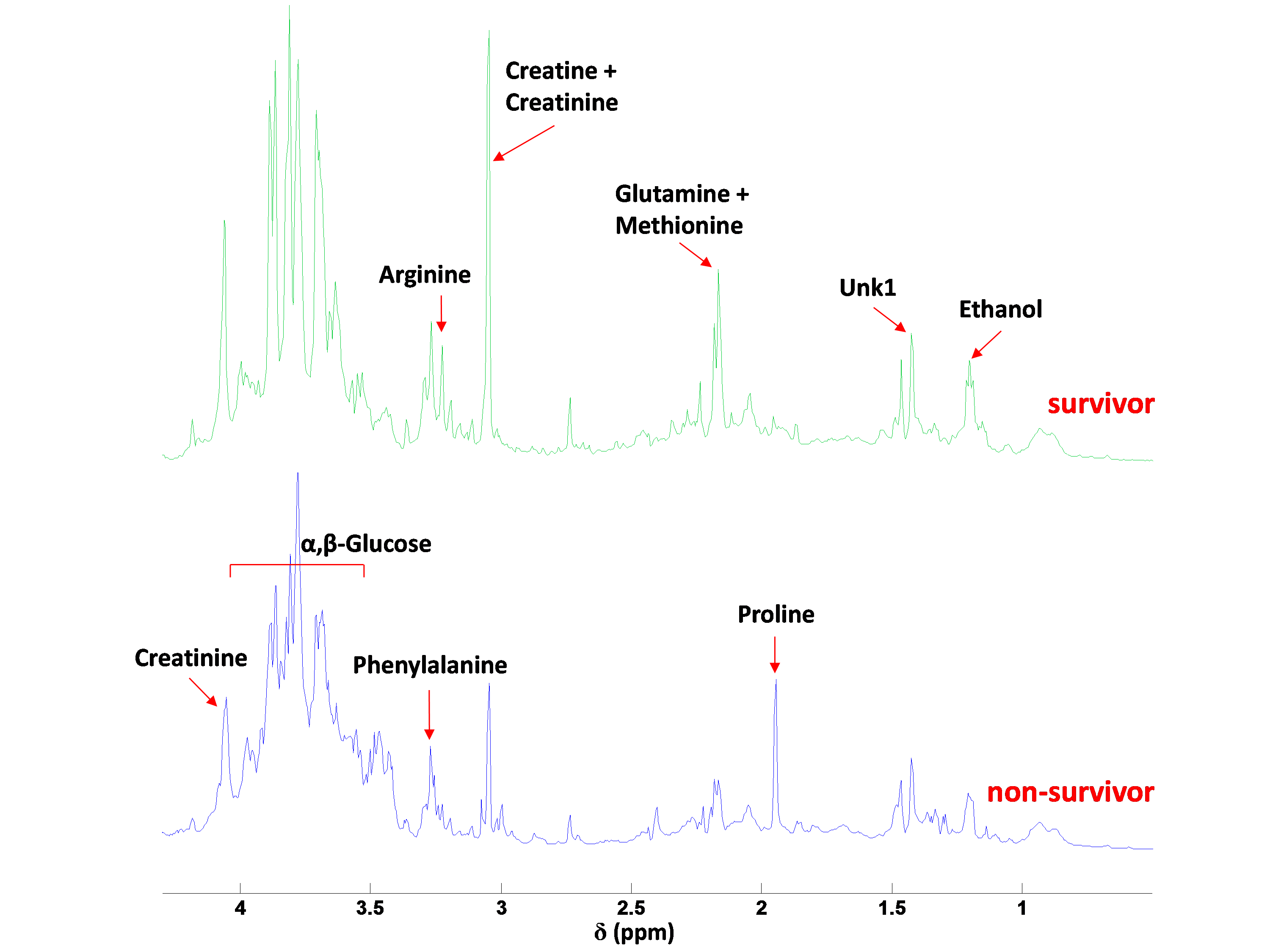

Supplement: S1 Fig — Comparison between survivor (green) and non-survivor (blue) patients’ metabolomic profile and assignation of different metabolites. (TIF) [file pone.0140993.s001.tif]

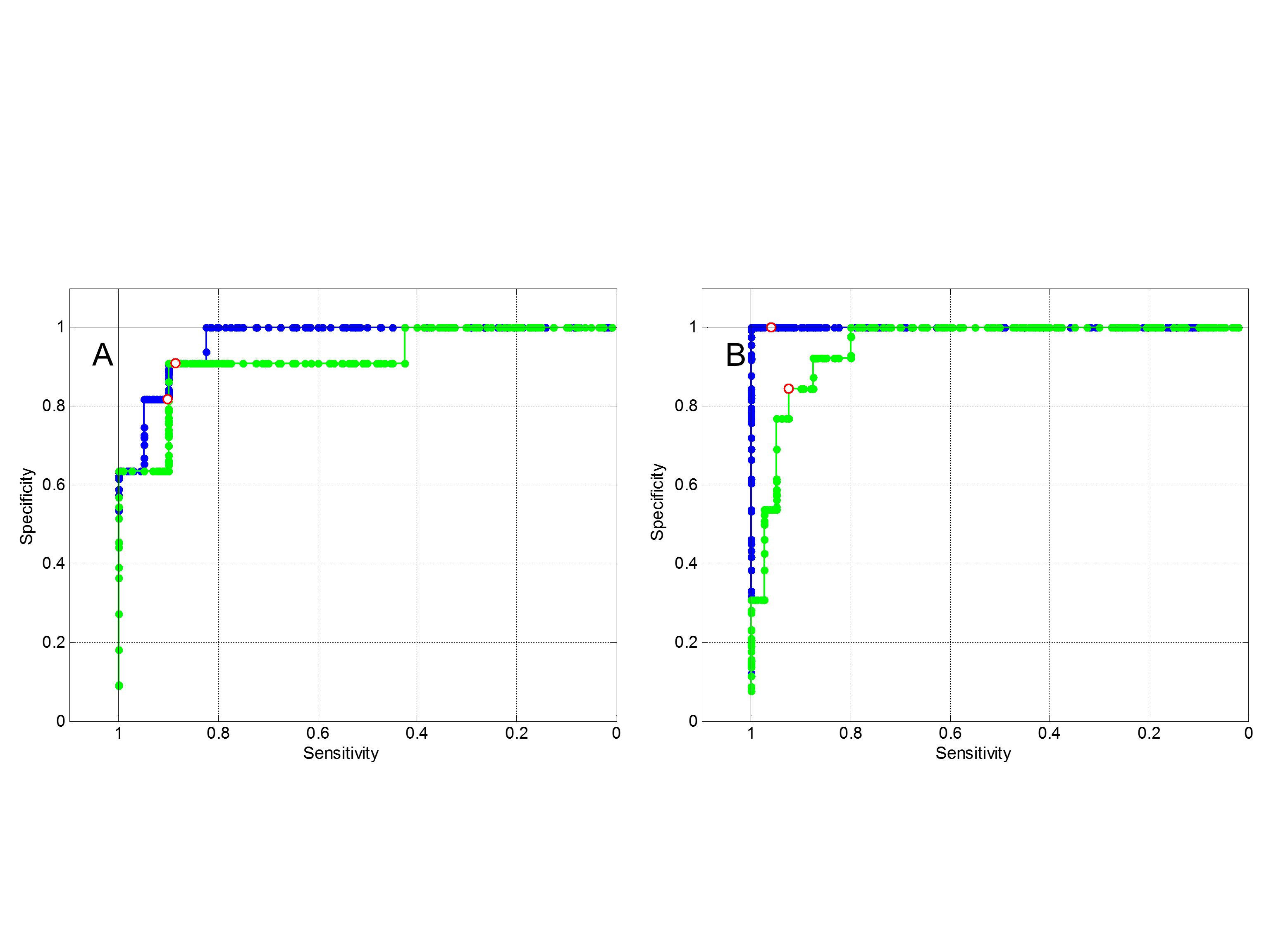

Supplement: S2 Fig — (TIF) [file pone.0140993.s002.tif]

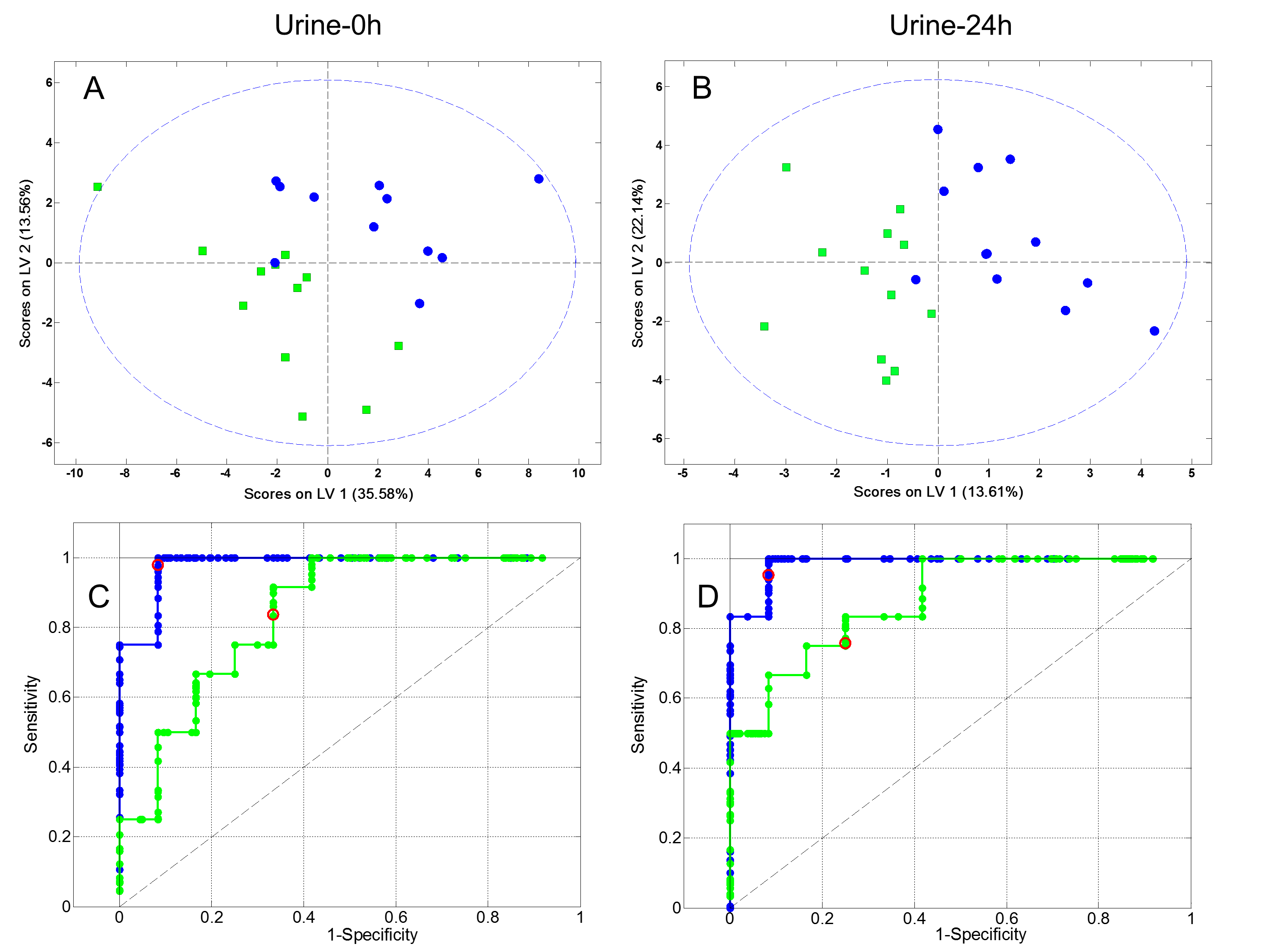

Supplement: S3 Fig — A) PLS-DA score plot constructed with Urine-0h samples obtained at the admission to the ICU and B) PLS-DA score plot constructed with Urine-24h samples obtained at 24h after admission to the ICU. ROC curves for discrimination between survivor and non-survivor patients constructed with C) Urine-0h and D) Urine-24h samples based on the matched PLS-DA model (blue line, training data; green line, leave-one-out cross-validation). (TIF) [file pone.0140993.s003.tif]
