# Supplementary material for: Prognosis Biomarkers of Severe Sepsis and Septic Shock by 1H NMR Urine Metabolomics in the Intensive Care Unit
Source: PLoS One. 2015 Nov 13;10(11):e0140993. doi: 10.1371/journal.pone.0140993 (PMC4643898; doi:10.1371/journal.pone.0140993)
Supplement: S1 Table — (DOCX) [file pone.0140993.s004.docx]

**Title: “Prognosis biomarkers of severe sepsis and septic shock by ^1^H NMR urine Metabolomics in the ICU”.**

**S1 Table:** Short summary of the medication record for the time-frame of sample collection. For each group, the number of patients to whom was given the medication is indicated.

| **Antibiotic** | | **Non-survivor** | **Survivor** |
| --- | --- | --- | --- |
| Quinolone | LEVOFLOXACIN(500 mg/24 h) | 4 | 28 |
|  | CIPROFLOXACIN (200 mg /12 h) | 1 | 1 |
| Cephalosporin | CEPHALOSPORINS 3^Th^ | 4 | 21 |
| Carbapenems | MEROPENEM (1 gr /6 h) | 3 | 6 |
|  | IMIPENEM (500 mg /6 h) | 0 | 4 |
| Penicillins | PIPERACILLIN TAZOBACTAM (4,5 g /6 h) | 4 | 13 |
|  | AMPICILLIN (3 gr/6 h) | 0 | 2 |
| Tetracyclines | DOXYCYCLINE (100 mg/12 h) | 1 | 0 |
| Oxazolidinones | LINEZOLID (600 mg /12 h) | 3 | 0 |
| Antiviral | OSELTAMIVIR (75 mg /12 h, oral) | 2 | 16 |
|  | ACICLOVIR (200 mg/4 h ,oral) | 1 | 1 |
| Aminoglycosides | GENTAMICIN (3 mg/kg/day) | 0 | 1 |
|  | AMIKACIN (20 mg/Kg/day) | 3 | 2 |
|  | CLARITHROMYCIN (500 mg/12 h) |  |  |
| Macrolides | AZITHROMYCIN (500 mg/day) | 2 | 8 |
| Nitroimidazoles | METRONIDAZOLE (500 mg/ 8 h) | 1 | 0 |
| Glycopeptides | VANCOMYCIN (1000 mg /12 h) | 0 | 2 |
| Glycylcyclines | TIGECYCLINE (50 mg/12 h) | 0 | 1 |
| Echinocandins | VORICONAZOLE (400 mg/12 h) | 1 | 1 |
|  | CASPOFUNGIN (50 mg /day) | 3 | 0 |
