# Supplementary material for: Prognosis Biomarkers of Severe Sepsis and Septic Shock by 1H NMR Urine Metabolomics in the Intensive Care Unit
Source: PLoS One. 2015 Nov 13;10(11):e0140993. doi: 10.1371/journal.pone.0140993 (PMC4643898; doi:10.1371/journal.pone.0140993)
Supplement: S2 Table — (DOCX) [file pone.0140993.s005.docx]

**Title: “Prognosis biomarkers of severe sepsis and septic shock by ^1^H NMR urine Metabolomics in the ICU”.**

**S2 Table:** Metabolite levels in survivor and non-survivors groups.

| **Metabolite dominant in the spectral region** | **Urine-0h survivors** | **Urine-0h Non-survivors** | ***p value** | **Urine-24h Survivors** | **Urine-24h Non-survivors** | ***p value** |
| --- | --- | --- | --- | --- | --- | --- |
|  |  |  |  |  |  |  |
| Ethanol | 9.5 ± 0.9 | 14 ± 2 | 0.036 | 11 ± 1 | 17 ± 3 | 0.0185 |
| Unknown | 7.2 ± 0.9 | 11 ± 2 | 0.05 | 6.1 ± 0.5 | 12 ± 3 | 0.0098 |
| Methionine + Glutamine | 32 ± 3 | 18.5 ± 0.9 | 0.029 | 27 ± 3 | 23 ± 3 | 0.43 |
| Arginine | 10.3 ± 0.9 | 6.2 ± 0.8 | 0.016 | 13 ± 1 | 4.6 ± 0.5 | 0.0005 |
| Phenylalanine | 12 ± 1 | 5.7 ± 0.7 | 0.007 | 11.2 ± 0.8 | 6.1 ± 0.6 | 0.0011 |
| Glucose | 0.53 ± 0.06 | 1.6 ± 0.3 | 8,00E-05 | 0.33 ± 0.02 | 2.5 ± 0.7 | 0.0043 |
| Hippurate | 0.67 ± 0.07 | 3 ± 0.7 | 8,00E-05 | 0.9 ± 0.1 | 4 ± 1 | 0.0013 |

Differences between the metabolites in survivors and non-survivors groups are expressed as mean ± standard error. Intensity and error values have been multiplied by 10^3^ for a major clarity. *p value survivor *versus* non-survivor patients.
