# Supplementary material for: Prognosis Biomarkers of Severe Sepsis and Septic Shock by 1H NMR Urine Metabolomics in the Intensive Care Unit
Source: PLoS One. 2015 Nov 13;10(11):e0140993. doi: 10.1371/journal.pone.0140993 (PMC4643898; doi:10.1371/journal.pone.0140993)
Supplement: S3 Table — (DOCX) [file pone.0140993.s006.docx]

**Title: “Prognosis biomarkers of severe sepsis and septic shock by ^1^H NMR urine Metabolomics in the ICU”.**

**S3 Table**: This table shows two examples of cases where there is a significant disparity between the prediction of SOFA scale and the metabolomic score. In these two distinct situations, metabolomics approach seems to be more efficient than the SOFA scores in the early prognosis of death.

| **Items** | **Patient type 1** | | **Patient type 2** | | **All (n=60)** | **Survivor (n=48)** | **Non-survivor (n=12)** | ***P value** |
| --- | --- | --- | --- | --- | --- | --- | --- | --- |
| Male sex, n (%); [IC] | Male | Female | Male | Female | 39 (65%); [53.6-77.8] | 32 (66.7%) | 7 (58.3%) | ns |
| Age, years, median (IQR); [IC] | 61 | 39 | 67 | 45 | 60 (47-73); [55.3-62.8] | 60 (24-80) | 65 (37-79) | ns |
| Severe sepsis, n (%); [IC] |  |  |  | Yes | 30 (50%); [38-63.5] | 27 (56.2%) | 3 (25%) | ns |
| Septic shock, n (%); [IC] | Yes | Yes | Yes |  | 30 (50%); [36.5-62] | 21 (43.8%) | 9 (75%) | ns |
| Days in the ICU, median (IQR); [IC] | 9 | 3 | 6 | 20 | 7 (4-11); [5-9] | 6.5 (4.7-11) | 7 (3-22.5) | ns |
| APACHE II, mean ± SD; [IC] | 21 | 21 | 13 | 20 | 19.6 ± 6.0; [18.1-21.1] | 19 ± 6 | 21 ± 5 | ns |
| SOFA-0h, median (IQR) | 8 | 7 | 13 | 11 | 8 (6.8-8.6) | 7 (5-10) | 11 (7-13.5) | < 0.05 |
| SOFA-24h, median (IQR) | 7 | 8 | 10 | 9 | 5 (3-8) | 5 (3-7) | 8 (4.75-9.75) | < 0.05 |
| SOFA-72h, median (IQR) | 7 | - | 5 | 8 | 3 (2-6) | 3.5 (2-5) | 6.5 (2-10.25) | < 0.05 |
| Origin of sepsis, n (%); [IC]: |  |  |  |  |  |  |  |  |
| -  Abdominal | X | X |  |  | 7 (11.7%); [2.4-18] | 6 (12.5%) | 1 (8.3%) | ns |
| -  Pulmonary |  |  | X | X | 38 (63.3%); [52-76.4] | 31 (64.6%) | 7 (58.3%) | ns |
| -  Urinary |  |  |  |  | 8 (13.3%); [4.5-22.3] | 7 (14.6%) | 1 (8.3%) | ns |
| -  CNS |  |  |  |  | 4 (6.7%); [1.7-14.6] | 4 (8.3%) | 0 | ns |
| Bacteremia, n (%); [IC] | X | X | X | X | 22 (36.7%); [23.6-48] | 14 (29.2%) | 8 (66.7%) | <0.05 |
| Metabolomic Score | | | | |  |  |  |  |
| Urine-0h | B.P. | B.P. | B.P. | B.P. |  |  |  |  |
| Urine-24h | B.P. | B.P. | B.P. | B.P. |  |  |  |  |

*ICU*, intensive care unit; *SOFA*, sequential organ failure assessment; SOFA-0h, score at admission; SOFA-24h, score 24 h after admission to the ICU; SOFA-72h, score 72 h after admission to the ICU; *CNS*, central nervous system; *B.P.*, bad prognosis in metabolomic score; *ns*, no significant value (p ≥ 0.05).
