# Supplementary material for: Prognosis Biomarkers of Severe Sepsis and Septic Shock by 1H NMR Urine Metabolomics in the Intensive Care Unit
Source: PLoS One. 2015 Nov 13;10(11):e0140993. doi: 10.1371/journal.pone.0140993 (PMC4643898; doi:10.1371/journal.pone.0140993)
Supplement: S1 Text — (DOCX) [file pone.0140993.s007.docx]

**Title: “Prognosis biomarkers of severe sepsis and septic shock by ^1^H NMR urine Metabolomics in the ICU”.**

**Mónica García-Simón, MD^1,#^; José M. Morales, PhD^2,#^; Vicente Modesto-Alapont, MD, PhD^3^; Vannina G. Marrachelli, PhD^4^; Rosa Vento Rehues, MD^1^; Angela Jordá Miñana, MD^1^; José Blanquer-Olivas, MD, PhD^1^; Daniel Monleón, PhD^4^.**

**^1^Department of Critical Care, Clinical University Hospital of Valencia, Valencia, Spain.**

**^2^Central Unit of Research in Medicine, University of Valencia, Valencia, Spain.**

**^3^Department of Paediatric Critical Care, University and Polytechnic Hospital La Fe, Valencia, Spain.**

**^4^Clinical Hospital Research Foundation-INCLIVA, Valencia, Spain.**

**# These authors contributed equally to this work.**

**METHODS**

**Sample collection**

As part of the daily clinical routine, for each patient were measured and recorded the values of haemoglobin (g/dl), leukocytes (x10^9^/l), platelets (x10^9^/l), glucose (mg/dl), creatinine (mg/dl), urea (mg/dl), bilirubin (mg/dl), C-reactive protein (CRP) (mg/l), procalcitonin (PCT) (ng/ml) and lactate (mmol/l). The study included the assessment of demographic information, admission diagnosis (severe sepsis/septic shock), origin of sepsis, days of ICU stay, Acute Physiology and Chronic Health Evaluation (APACHE II) score (1) at admission, bacteremia in the blood culture and mechanical ventilation. This information was managed using an Excel database (Microsoft Excel 2008), SPSS version 20 (IBM Statistical Packaged for social sciences software, MAC) and Epidat 3.1 (Epidemiological Dates Analysis, Galicia, Spain).

**^1^H NMR Spectroscopy and metabolite concentration profiling**

The total preparation time for each sample before NMR spectroscopy was less than 15 min. Preparation consisted of the addition of 40µl of a phosphate buffer solution (0.06M Na_2_HPO_4_/ 0.04M NaH_2_PO_4_, pH 7) and 40µl of sodium-3´-trimethylsilylpropionate-2,2,3,3-d_4_ (TSP, 0.5 mM) in deuterium oxide (D_2_O) to 420µl of urine. Sample placed in a 5mm high-resolution NMR tube. All spectra were recorded in a Bruker Avance DRX 600 spectrometer (Bruker GmbH, Rheinstetten, Germany). Nominal temperature of the sample was kept at 310K. Lock homogeneity was achieved by extensive manual coil-shimming using one-dimensional (1D) water pre-saturation experiments in interactive mode. Visual lineshape inspection of the alanine doublet signal at 1.475 ppm was used for lock homogeneity shimming.

A single-pulse pre-saturation experiment was acquired in all samples. To avoid effects on signal intensity different from metabolite amount, unfiltered single-pulse experiments were preferred over filtered pulse sequences, such as Carr–Purcell–Meiboom–Gill or nuclear Overhauser effect spectroscopy. A total of 64 transients were collected into 64k data points for all the experiments. Water pre-saturation for 1 s along the recycling delay was used for solvent signal suppression. The spectral width for all spectra was 14 ppm. Before Fourier transformation, the free induction decay was multiplied by a 0.3 Hz exponential line broadening.

All spectra were phased, baseline corrected carefully and chemical shifts were adjusted with reference to TSP signal at 0 ppm using MestReNova 6.2 software (Mestrelab Research S.L., Santiago de Compostela, Spain). The spectra were binned into 0.005 ppm buckets between 0.5-10 ppm and mean centred for multivariate analysis and normalized to total aliphatic spectral area (0.5-4.4 ppm) to eliminate differences in metabolite total concentration. Data were imported into MATLAB R2012a (The MathWorks Inc., Natick, MA 2012) for additional processing and further analysis. Signals belonging to selected metabolites were integrated and quantified using semi-automated in-house MATLAB peak-fitting routines.

**Statistical modelling. Metabolomic mortality model.**

Chemometrics statistical analyses were performed using in-house MATLAB scripts and the PLS Toolbox 6.7 (Eigenvector Research, Inc., Wenatchee, WA, USA). Metabolite levels were expressed as mean ± SD (standard deviation). One-way-analysis of variance (ANOVA) was used in order to determinate the statistical significance between the means in both survivor and non-survivor groups. Principal component analysis (PCA) and projection to latent structures for discriminant analysis (PLS-DA) were applied to NMR spectral datasets (2). PCA is able to find low dimensional embedding of multivariate data, in a way that optimally preserves the structure of the data. PCA technique transforms variables in a data set into a smaller number of new latent variables called principal components (PCs), which are uncorrelated to each other and account for decreasing proportions of the total variance of the original variables. Each new PC is a linear combination of the original variation such that a compact description of the variation within the data set is generated. Observations are assigned scores according to the variation measured by the principal component with those having similar scores clustering together.

Where PCA proved inadequate to define clustering, a supervised approach was used. PLS-DA is a supervised extension of PCA used to distinguish two or more classes by searching for variables (X matrix) that are correlated to class membership (Y matrix). In this approach the axes are calculated to maximize class separation and can be used to examine separation that would otherwise be across three or more principal components (3).

**Comparison with conventional SOFA score.**

The PLS-DA model discriminating between controls and patients was cross-validated by the leave-one-out method. Cross-validation is a very useful tool that provides two critical functions in Chemometrics. First, cross validation enables an assessment of the optimal complexity of a model. Second, it allows an estimation of the performance of a model when it is applied to unknown data. Leave-one-out cross-validation involves the use of a single observation from the original sample as the validation data, and the remaining observations as the training data. This is repeated in a manner that each observation in the sample is used once as the validation data.

To compare performance in the mortality prediction of Metabolomic and SOFA scores a multi-step statistical analysis was performed based on the study of Cabré et al.(4) Continuous data are presented as means and standard deviations or medians and interquartile ranges and when appropriate were compared for statistical differences using the unpaired t or Mann-Whitney U test. Categorical data are reported as proportions rounded to the nearest whole number and where applicable were tested for significance using the χ2 or Fisher exact test. To study the relationship between the SOFA scores and mortality, we have calculated for each patient a linear regression determined by the intercept in origin (SOFA-0h) and the slope (evolutionary value SOFA-24h and -72h). The same procedure was followed with metabolomics scores. In this case, the intercept in origin was Urine-0h and the slope, Urine-24h. With the values obtained, we fitted a multivariable logistic regression model using the sequential method stepwise forward base on the likelihood ratio (entry p ≤ 0.05) to see if the findings are significant for predicting death.

To see that the model is a good diagnostic method we have calculated the calibration using the analysis of the goodness of fit, using the statistical technique described by Hosmer-Lemeshow. To calculate the discrimination we have obtained the area under the ROC curve and diagnostic quality indices (sensitivity, specificity, positive and negative predictive values, likelihood ratio) for the optimal cut-off. In order to compare the results of both ROC curves, Metabolomics and SOFA, we used the test of Hanley and McNeil. In all statistical tests, p≤ 0.05 was considered to indicate statistical significance.

**REFERENCES**

S1. Knaus WA, Draper EA, Wagner DP, Zimmerman JE. APACHE II: a severity of disease classification system. Crit Care Med 1985; 13: 818-829.

S2. Verboven, S. and M. Hubert; LIBRA: a MATLAB library for robust analysis. Chemometrics and Intelligent Laboratory Systems 2005; 75: 127-136.

S3. Trygg J, Holmes E, Lundstedt T. Chemometrics in metabonomics. J Proteome Res 2007; 6:469-479.

S4. Cabré L, Mancebo J, Solsona JF, Saura P, Gich I, Blanch L, Carrasco G, Martín MC, Working group of the SEMICYUC. Multicenter study of the multiple organ dysfunction syndrome in intensive care units: the usefulness of Sequential Organ Failure Assessment scores in decision making. Intensive Care Med 2005; 31: 927-933.
